# Endobronchial fibroepithelial polyp presenting as persistent wheezing in a 9-year-old girl: a case report

**DOI:** 10.3389/fped.2026.1938806

**Published:** 2026-09-15

**Authors:** Veronica Ferasin, Francesco Sansone, Stefania Zanconato, Cesare Cutrone, Marianna Sari, Valentina Agnese Ferraro, Silvia Carraro

**Affiliations:** 1 Women’s and Children’s Health Department - SDB, University of Padova, Padova, Italy; 2 Unit of Pediatric Allergy and Respiratory Medicine, University Hospital of Padova, Padova, Italy; 3 Unit of Pediatrics, Rovigo Hospital, Rovigo, Italy; 4 Department of Otolaryngology, Azienda Ospedale Università di Padova, Padova, Italy

**Keywords:** bronchiectasis, endobronchial tumour, fibroepithelial polyp, paediatrics, wheezing

## Abstract

Fibroepithelial polyps are benign tumours that usually arise from cutaneous or mucosal surfaces. In children they most commonly involve the skin, oral cavity, and genitourinary tract, whereas endobronchial localization is exceptionally rare. We report the case of a 9-year-old girl with persistent biphasic wheezing, cough and dyspnoea on exertion despite appropriate asthma-directed treatment. High-resolution computed tomography of the chest revealed an endobronchial lesion associated with distal bronchiectatic changes, and bronchoscopy confirmed the presence of a protruding endobronchial polyp. The lesion was completely removed endoscopically, and histopathological and immunohistochemical examination confirmed the diagnosis of a benign fibroepithelial polyp. Following removal, the respiratory symptoms resolved completely, with substantial regression of the bronchiectatic changes at six-month follow-up. This case highlights the importance of considering structural airway obstruction in children with persistent or biphasic wheezing that fails to respond to appropriate asthma therapy.

## Introduction

Primary pulmonary neoplasms are rare in children, accounting for approximately 0.2% of all primary paediatric tumours (1). Endobronchial tumours are even rarer, with no definitive epidemiological data.

Carcinoid tumours are the most frequently diagnosed, accounting for 80% of all primary pulmonary neoplasms in children (2). Fibroepithelial polyps are benign tumours usually located on skin, oral cavity or genitalia (3). To the best of our knowledge, only few cases of endobronchial fibroepithelial polyps are described in literature and only one in a paediatric patient (3).

## Case presentation

A previously healthy 9-year-old girl was referred to our centre for persistent wheezing, cough, and exertional dyspnoea (Figure 1).

**Figure 1:**
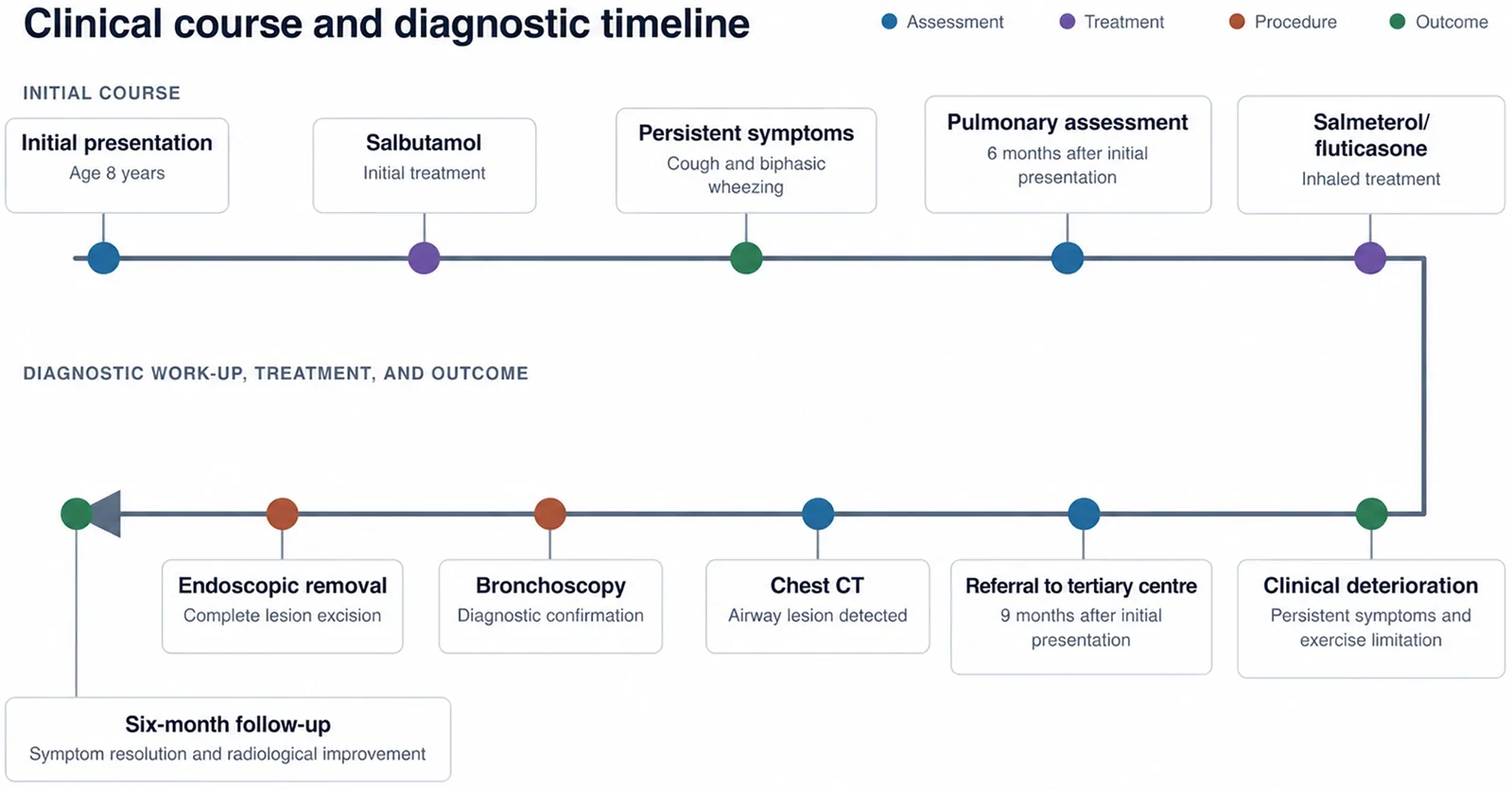
Timeline illustrating the patient’s clinical course.

She was born at term after an uneventful pregnancy and perinatal period, with normal psychomotor development and vaccinations reported as up to date. She remained well during the first years of life. At the age of 6, she experienced a left basal pneumonia, treated with amoxicillin. Her family history was notable for asthma in her grandfather, with no other relevant family history of respiratory or allergic diseases. She had no personal history of atopy and no exposure to passive smoking or household pets.

At 8 years of age, wheezing was first detected on chest auscultation by her primary care paediatrician. There was no history of choking episodes or suspected foreign-body aspiration. No nocturnal symptoms or episodes of haemoptysis were reported. Treatment with as-needed salbutamol was initiated but resulted in no significant clinical improvement. A chest x-ray was performed, showing no pathological signs. Over the following 5 months, respiratory symptoms followed an intermittent-relapsing course, with no clear response to salbutamol, inhaled corticosteroids or antibiotics. Six months after the initial onset of respiratory symptoms, the patient developed persistent cough and biphasic wheezing, audible during both inspiration and expiration and consistently documented at each clinical assessment, prompting referral to a paediatric pulmonologist. At evaluation, her weight was 30 kg, axillary temperature was 36.5 °C, heart rate was 85 beats/min, respiratory rate was 18 breaths/min, blood pressure was 105/65 mmHg, and peripheral oxygen saturation was 100% while breathing room air. She appeared clinically well, with no signs of respiratory distress, chest retractions, or use of accessory respiratory muscles. Chest expansion was symmetrical and air entry was preserved bilaterally. Diffuse inspiratory and expiratory wheezing was the only abnormal finding on respiratory examination; no crackles or other added sounds were detected. Skin prick tests were negative for aeroallergens. Baseline spirometry showed reduced FEV₁ (66% of predicted) and FVC (63% of predicted), with a preserved FEV₁/FVC ratio (82.7%) and a reduced FEF₂₅–₇₅ (52% of predicted). Predicted values were calculated using the Global Lung Function Initiative reference equations endorsed by the European Respiratory Society (4). Although the findings were initially considered suggestive of a possible mixed restrictive-obstructive pattern, a restrictive defect could not be confirmed because static lung volumes were not measured. In the suspicion of bronchial asthma, maintenance therapy with inhaled salmeterol/fluticasone was initiated, with adequate adherence reported. After 3 months of treatment, spirometry showed FEV₁ of 81% of predicted and FVC of 99% of predicted, with a reduced FEV₁/FVC ratio (70%) and FEF₂₅–₇₅ (39%). The bronchodilator response was negative, with an increase in FEV₁ of only 4%. These findings were interpreted as an obstructive ventilatory pattern with a disproportionate reduction in mid-expiratory flow. Although FEV₁ had increased from 66% to 81% of predicted, the persistent airflow obstruction, the absence of a significant bronchodilator response, the lack of clinical improvement and the development of exertional dyspnoea prompted further diagnostic investigation and the girl was referred to our centre (Figure 1).

At our evaluation clinical and functional characteristics raised concern for a fixed or structural airway obstruction rather than poorly controlled asthma. The main differential diagnosis included foreign- body aspiration, extrinsic airway compression, mucus obstruction, and benign or malignant endobronchial lesions. Further investigations were warranted including a chest high-resolution computed tomography (HRCT) and a flexible bronchoscopy to assess both lung parenchyma and tracheobronchial anatomy. Chest HRCT was performed, showing an exophytic mass of 8 × 10 × 16 mm inside the right endobronchial tree, hanging from the bifurcation between the upper-lobe bronchus and the intermediate bronchus (Figure 2). Cylindrical bronchiectasis were found in the middle and lower lobes, associated with segmental parenchymal consolidation (Figure 3A). Flexible bronchoscopy, performed under general anaesthesia, confirmed the presence of a pedunculated polyp arising at the bifurcation between the right upper-lobe bronchus and the bronchus intermedius, causing partial obstruction of the bronchus intermedius; the percentage of luminal obstruction was not formally quantified. The lesion had a smooth, non-lobulated, reddish surface, firm consistency and minimal superficial vascularity (Figure 4). After visualising it with the flexible bronchoscope, the polyp was removed endoscopically using a rigid bronchoscope (outer 6 mm, length 30 cm) (KARL STORZ SE & Co. KG, Tuttlingen, Germany) equipped with a conchotome with a 4 mm, 0-degree endoscope, connected to an Olympus HD video tower (Olympus, Hamburg, Germany). No laser or other thermal energy devices were required for haemostasis. The procedure lasted about 20 min. No intraoperative or post-procedural complications occurred. Microscopically the lesion showed a benign biphasic architecture composed of bronchial-type epithelium and a hypocellular stromal component with mild lymphomonocytic and granulocytic inflammation. No significant cytological atypia, increased mitotic activity, or tumour necrosis was identified. Immunohistochemically, the epithelial component was positive for cytokeratin 7, whereas the mesenchymal component showed focal positivity for smooth muscle actin and desmin. Myf4, ALK-D5F3, ROS1, TTF-1, oestrogen receptor, and progesterone receptor were negative. Biphasic morphology, absence of malignant histological features, and immunophenotypic profile supported the exclusion of other epithelial and mesenchymal neoplasms. Overall, the findings were consistent with a benign fibroepithelial neoplasm, specifically an adenofibroma.

**Figure 2:**
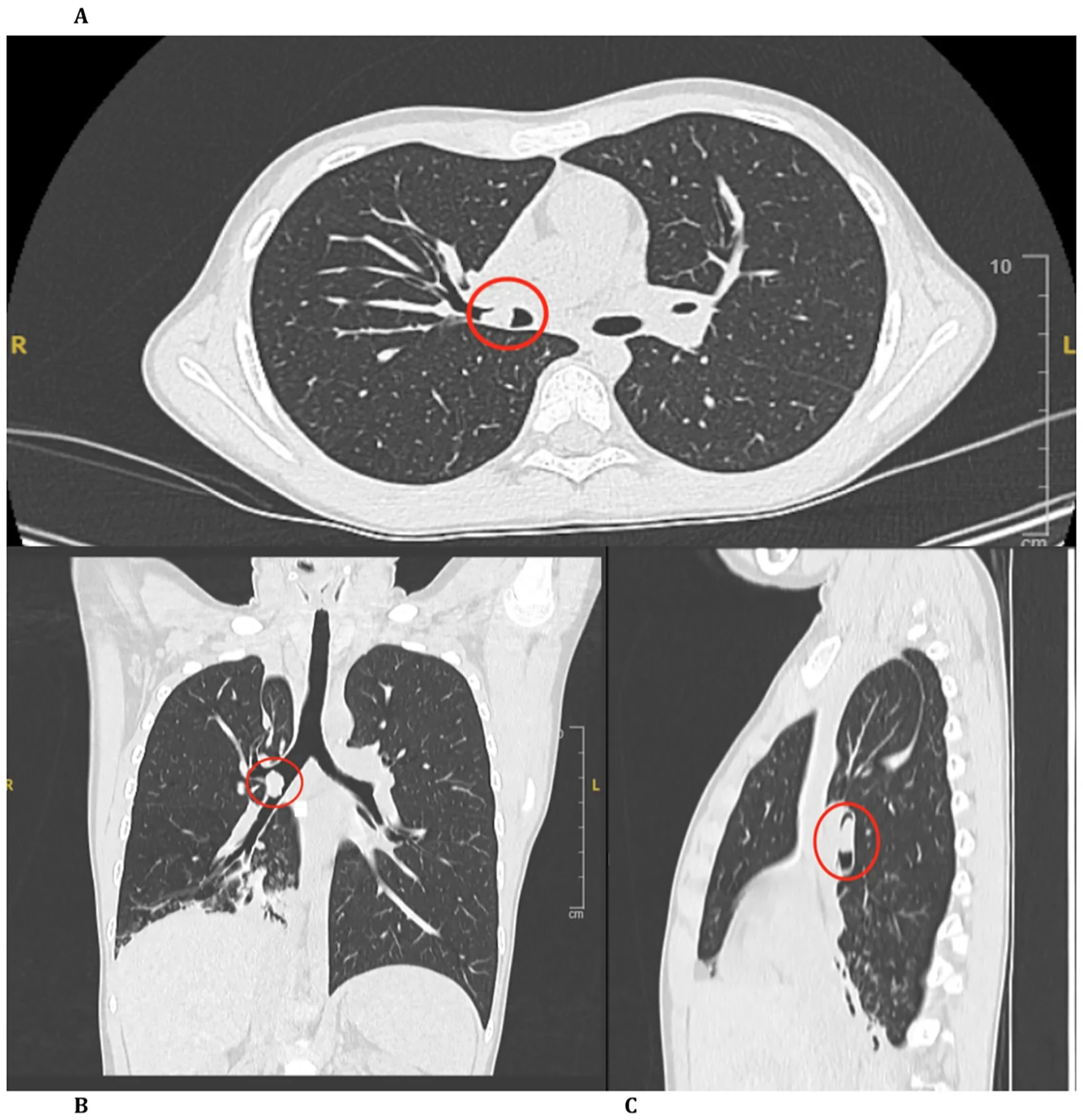
Axial **(A)**, coronal **(B)**, and sagittal **(C)** reconstructions of the chest high resolution computed tomography (HRCT) scan demonstrating an exophytic endobronchial mass hanging from the bifurcation between the upper-lobe bronchus and the intermediate bronchus.

**Figure 3:**
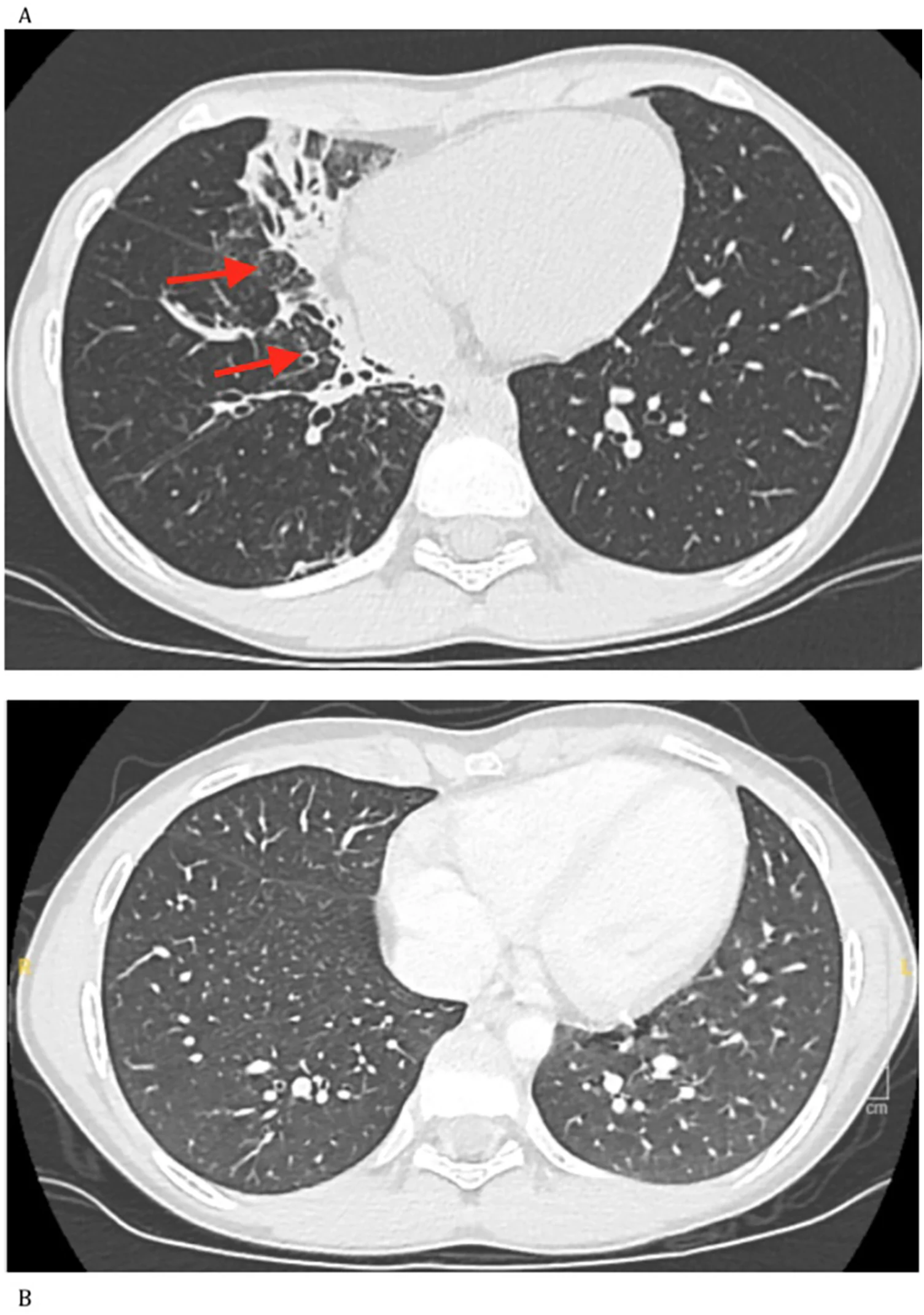
**(A)** Axial reconstructions of the chest HRCT scan performed at the time of diagnosis, demonstrating cylindrical bronchiectasis in the middle and lower lobes (arrows), with segmental parenchymal consolidation. **(B)** Axial reconstructions of the chest HRCT scan performed 6 months after endoscopic treatment, demonstrating almost complete regression of bronchiectasis in the middle and lower lobes.

**Figure 4:**
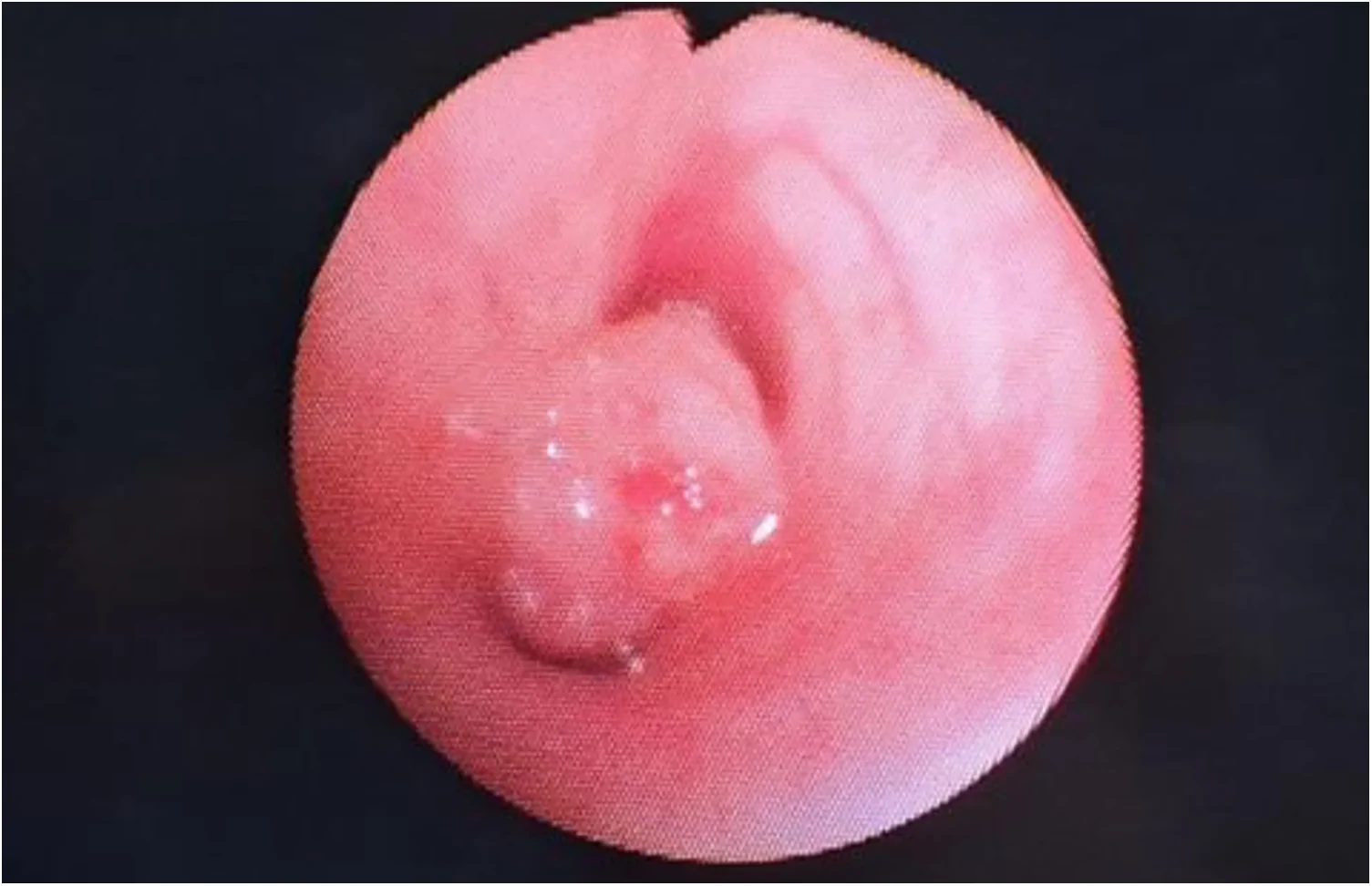
Bronchoscopy view demonstrating a polyp obstructing the right intermediate bronchus.

After the procedure the wheezing sound on chest auscultation disappeared and the patient showed a complete resolution of her symptoms. At the six-month follow-up, the patient remained asymptomatic, with no recurrence of wheezing or respiratory infections. She had discontinued all inhaled asthma medications and did not require any further respiratory treatment. Follow-up spirometry showed normal lung function, with FEV₁ of 97% of predicted, FVC of 97% of predicted, FEV₁/FVC ratio of 92%, and FEF₂₅–₇₅ of 113% of predicted.

Chest HRCT and flexible bronchoscopy showed no sign of tumour relapse and almost complete regression of bronchiectasis in the middle and lower lobes (Figure 3B).

## Discussion

We presented the case of a young girl with an endobronchial fibroepithelial polyp. This kind of benign tumour is rarely localized in the bronchial tree, occurring most of the time on skin, oral cavity or genito-urinary tract. To the best of our knowledge, the only paediatric case of endobronchial fibroepithelial polyp in the literature has been described by Thimmesch in 2022 (3). The patient was a 14 years-old boy admitted for epigastralgia with asymmetric chest expansion and reduced breath sounds in the left lung. CT scan showed partial atelectasis of the left lung and spirometry highlighted a restrictive pattern. The polyp was detected by means of flexible bronchoscopy and removed endoscopically. Compared with the case reported by Thimmesch et al., our patient was younger (8 vs. 14 years) and presented predominantly with persistent biphasic wheezing while the previous case was characterized by asymmetric chest expansion and reduced breath sounds in the left lung with no added pathologic sounds. Interestingly, both patients had exertional dyspnoea. Our patient had a smaller right-sided polyp (8 × 10 × 16 mm) partially obstructing the intermediate bronchus, while the larger lesion described by Thimmesch et al. (35 × 15 × 10 mm) completely obstructed the left main bronchus, justifying the lack of sounds on auscultation. The associated radiological findings were cylindrical bronchiectasis and segmental consolidation in our case, compared with partial left-lung atelectasis without reported bronchiectasis. Both patients had abnormal pulmonary function initially attributed to asthma and underwent successful rigid bronchoscopic resection, although electrocautery was required in the previously reported case. Both children experienced complete clinical recovery without recurrence.

Symptoms associated with endobronchial tumours are usually non-specific, including recurrent dyspnoea, fatigue, persistent wheezing non-responsive to bronchodilators, and recurrent pneumonia (5). As in our case, it happens frequently that patients are labeled as asthmatic and treated for months to years with inhaled corticosteroids and bronchodilators with poor response (6). The persistence of respiratory symptoms and spirometry alterations despite appropriate treatment should always lead to consider alternative diagnosis, as highlighted by Global Initiative for Asthma (GINA) recommendations (7).

Diagnostic workup should always include a chest CT-scan and a bronchoscopy. Chest x-ray can show signs of pulmonary consolidation due to atelectasis, but its sensitivity is low, resulting normal in 10% of patients (5). Chest CT and bronchoscopy have complementary diagnostic roles. Even though endobronchial masses may not always be clearly identified on radiological imaging, CT is recommended to accurately assess the extention of the lesion, its relationship with the surrounding anatomical structures, the involvement of the distal lung parenchyma and adjacent tissues, as well as the presence of potential metastatic disease (8).

Bronchoscopy, on the other hand, remains the diagnostic gold-standard, as it enables direct visualization and precise anatomical localization of the lesion, accurate assessment of its morphological features and of the degree of airway obstruction; furthermore, it allows tissue sampling for histopathological characterization and, in selected cases, may also serve as a definitive endoscopic therapeutic approach (2). Biopsies can be obtained using a flexible bronchoscope, while endoscopic exeresis requires a rigid one (9). Pathologic examination reveals the characteristics of the mass and allows its classification. Immunohistochemistry can help to further characterize the tumour when microscopic appearance is not sufficient. In our case the positivity for cytokeratin 7 in the outer layer of the polyp was consistent with an epithelial origin, while the positivity for desmin and actin in the core confirmed its connective tissue component.

The endoscopic approach used to eradicate the mass in the case described was adopted on the basis of mass localization and its polypoid appearance, suitable for radical exeresis. The same technique has been adopted in the similar case described by Thimmesch (3). In the last 30 years bronchoscopic management has been widely used for the treatment of benign endobronchial tumours. Success rate ranges from 50% to 80% and complications (i.e., pneumothorax, pneumomediastinum, mediastinal emphysema, and bronchial lymph fistula) are rare. Endobronchial resection is not indicated in case of total obstruction of the tracheobronchial lumen, tumour extension beyond the tracheobronchial lumen and tumour malignancy potential (10). Nonetheless, the available literature is limited and refers to the management of benign endobronchial tumours in the general population. It should be noted that there are no consensus statements or guidelines about the management of endobronchial masses, and of fibroepithelial polyps in particular, in children; thus the therapeutic decision should be based on patient’s characteristics, tumour presentation and past experience of the surgical team.

In the literature, no malignant transformation, metastasis or local relapse are reported following radical exeresis of a fibroepithelial polyp. Anyway, given the rarity of endobronchial polyps in children, the patient was re-evaluated 6 months after the endoscopic treatment by means of chest HRCT and bronchoscopy. Both exams were negative for pathological relapse. Moreover, the bronchiectatic changes had almost completely regressed, suggesting that paediatric bronchiectasis may be at least partially reversible when the underlying cause is identified and successfully treated (11). Early intervention, in fact, can stop the pathologic mechanism before the occurrence of structural non-modifiable changes (12). In our patient, the temporal association between endobronchial obstruction and the development of bronchiectasis suggests that impaired mucociliary clearance, mucus retention, recurrent infection and inflammation may have played a role in bronchiectasis development. Removal of the obstructing polyp likely interrupted this pathogenic cycle and was associated with almost complete regression of the bronchiectatic changes at the six-month follow-up.

## Conclusion

We presented an extremely rare case of endobronchial fibroepithelial polyp in a paediatric patient. As in other endobronchial masses clinical presentation was non-specific and characterized by recurrent or persistent respiratory symptoms, with little or no response to appropriate asthma-directed treatment. It is worth stressing that persistent respiratory symptoms or signs, not responding to an appropriate pharmacological treatment, should always be considered a red flag for possible underlying conditions and further diagnostic exams should be undertaken without waiting too long. Moreover, given the rarity of these tumours in the paediatric age, it is important to describe the clinical course of affected patients in order to improve the diagnostic and therapeutic management of these disorders.

## Data Availability

The original contributions presented in the study are included in the article/Supplementary Material, further inquiries can be directed to the corresponding author.
